# Oxfendazole mediates macrofilaricidal efficacy against the filarial nematode *Litomosoides sigmodontis in vivo* and inhibits *Onchocerca spec*. motility *in vitro*

**DOI:** 10.1371/journal.pntd.0008427

**Published:** 2020-07-06

**Authors:** Marc P. Hübner, Coralie Martin, Sabine Specht, Marianne Koschel, Bettina Dubben, Stefan J. Frohberger, Alexandra Ehrens, Martina Fendler, Dominique Struever, Edward Mitre, Nathaly Vallarino-Lhermitte, Suzanne Gokool, Sara Lustigman, Manfred Schneider, Simon Townson, Achim Hoerauf, Ivan Scandale

**Affiliations:** 1 Institute for Medical Microbiology, Immunology and Parasitology, University Hospital Bonn, Bonn, Germany; 2 Unité Molécules de Communication et Adaptation des Microorganismes (MCAM, UMR 7245), Sorbonne Universités, Muséum national d'Histoire naturelle, CNRS, Paris, France; 3 Drugs for Neglected Diseases *initiative*, Geneva, Switzerland; 4 Department of Microbiology and Immunology, Uniformed Services University of the Health Sciences, Bethesda, Maryland, United States of America; 5 Tropical Parasitic Diseases Unit, Northwick Park Institute for Medical Research, Harrow, London, United Kingdom; 6 Molecular Parasitology, New York Blood Center, New York, New York, United States of America; 7 Schneider Industrial Investment, PKPD&TOX Consulting, Amman, Jordan; 8 German Center for Infection Research (DZIF), partner site Bonn-Cologne, Bonn, Germany; University of Buea, CAMEROON

## Abstract

A major impediment to eliminate lymphatic filariasis and onchocerciasis is the lack of effective short-course macrofilaricidal drugs or regimens that are proven to be safe for both infections. In this study we tested oxfendazole, an anthelmintic shown to be well tolerated in phase 1 clinical trials. *In vitro*, oxfendazole exhibited modest to marginal motility inhibition of adult worms of *Onchocerca gutturosa*, pre-adult worms of *Onchocerca volvulus* and *Onchocerca lienalis* microfilariae. *In vivo*, five days of oral treatments provided sterile cure with up to 100% macrofilaricidal efficacy in the murine *Litomosoides sigmodontis* model of filariasis. In addition, 10 days of oral treatments with oxfendazole inhibited filarial embryogenesis in patent *L*. *sigmodontis*-infected jirds and subsequently led to a protracted but complete clearance of microfilaremia. The macrofilaricidal effect observed *in vivo* was selective, as treatment with oxfendazole of microfilariae-injected naïve mice was ineffective. Based on pharmacokinetic analysis, the driver of efficacy is the maintenance of a minimal efficacious concentration of approximately 100 ng/ml (based on subcutaneous treatment at 25 mg/kg in mice). From animal models, the human efficacious dose is predicted to range from 1.5 to 4.1 mg/kg. Such a dose has already been proven to be safe in phase 1 clinical trials. Oxfendazole therefore has potential to be efficacious for treatment of human filariasis without causing adverse reactions due to drug-induced microfilariae killing.

## Author summary

Onchocerciasis and lymphatic filariasis represent two debilitating filarial diseases that belong to the neglected tropical diseases. The current efforts to eliminate those diseases is hampered by the lack of short-course macrofilaricidal drugs, i.e. drugs that kill the adult worms, or regimens that are proven to be safe for both diseases. In the present study we demonstrate that the anthelmintic drug oxfendazole, currently used in veterinary medicine against intestinal helminths, has excellent efficacy in the *Litomosoides sigmodontis* rodent model of filariasis. Oxfendazole caused complete clearance of adult filariae after a short oral regimen *in vivo*. Oxfendazole was not directly active against the circulating filarial progeny, the microfilariae, suggesting that drug-induced serious adverse events due to the clearance of microfilariae are unlikely. Human dose was predicted based on the efficacy in the rodent model, the calculation estimated a low efficacious dose, which has already been shown to be safe in phase 1 clinical trials. Thus, oxfendazole represents a promising drug candidate for the treatment of human filarial diseases such as onchocerciasis and lymphatic filariasis.

## Introduction

Filarial nematodes cause debilitating diseases in tens of millions of people worldwide. Onchocerciasis causes vision impairment, blindness and dermatitis while lymphatic filariasis may cause lymphedema and hydrocele [1, 2]. Due to their prevalence and severe pathology, these infections have been selected by the World Health Organization (WHO) for elimination (onchocerciasis) and elimination as public health problem (lymphatic filariasis) by 2030 [3, 4].

Efforts to globally eliminate onchocerciasis and lymphatic filariasis are hampered by the absence of effective vaccines and a lack of adult-worm killing (macrofilaricidal) medications using a short course regimen. Current mass drug administration (MDA) programs rely only on microfilaricidal drugs that primarily kill microfilariae, the first stage larvae (L1) but not the adult worms when administered as annual or biannual single doses [2]. While recent studies suggest triple therapy with diethylcarbamazine (DEC), ivermectin, and albendazole may exert some macrofilaricidal activity against lymphatic filariasis [5], this combination is contraindicated in areas co-endemic for onchocerciasis or loiasis due to the potential risk of serious adverse effects caused by rapid microfilariae killing [6, 7].

Several well-known anthelmintic drugs belonging to the benzimidazole class have shown potent *in vivo* macrofilaricidal activity against filarial species in different animal models and were tested in patients with onchocerciasis in the past [8–10]. A detailed list of these studies is shown in S1 Table. Flubendazole and oxfendazole have excellent macrofilaricidal efficacy when administered subcutaneously (sc), while efficacy of flubendazole was reduced when used via the oral route (S1 Table [8, 11–14]). Although the efficacy of flubendazole against several filarial nematodes was validated in many studies [15–17], Janssen Pharmaceutica decided not to proceed with flubendazole in human clinical studies based on the toxicological profile [18]. Oxfendazole, on the other hand, was shown to be efficacious for the treatment of *Taenia solium* cysticercosis-infected pigs [19]. Subsequent investigational new drug program (IND) enabling studies provided the rationale to initiate a single ascending dose phase 1 study in humans [20]. A multiple ascending dose study using five daily doses of 3, 7.5 and 15 mg/kg was completed (https://clinicaltrials.gov/ct2/show/NCT03035760); the publication of results is pending. We anticipate that these oxfendazole tested dose ranges may also be sufficient to support a future proof of concept phase 2 clinical study for onchocerciasis.

In this study, we evaluated *in vitro* and *in vivo* using surrogate models of filariasis the efficacy of oxfendazole for the treatment of filarial infections. We first analyzed the *in vitro* activity of oxfendazole on bovine *Onchocerca lienalis* microfilariae, bovine *Onchocerca gutturosa* adult male worm and *Onchocerca volvulus* pre-adult worm motility. As humans are the only viable host for *O*. *volvulus*, drug discovery relies on surrogate parasites [21]. The *in vivo* efficacy of oxfendazole against filarial nematodes in this study was assessed using the rodent filarial nematode *Litomosoides sigmodontis* by both oral gavage and subcutaneous (sc) administration. The *L*. *sigmodontis* rodent model has been used to validate macrofilaricidal drug candidates by a consortium led by the Bill & Melinda Gates Foundation, Drugs for Neglected Diseases *initiative* (DND*i*), academia, and pharmaceutical companies and has supported the advancement of a few macrofilaricidal compounds through preclinical and clinical development [17, 22–28]. In this study we demonstrate that oxfendazole exhibits excellent macrofilaricidal efficacy following both oral and sc administrations in the *L*. *sigmodontis* model. Animal efficacious exposures are projected to be reached in humans at a clinically relevant and safe dose. DND*i* is thus exploring the possibility of repurposing oxfendazole as a macrofilaricidal treatment for filarial indications (https://www.dndi.org/diseases-projects/portfolio/oxfendazole/).

## Methods

### Ethics statement

All experimental procedures were conducted in accordance with EU Directive 2010/63/EU and the relevant national legislation, namely the French “Décret no 2013–118, 1er février 2013, Ministère de l’Agriculture, de l’Agroalimentaire et de la Forêt”, national license number 75–1415. Animal protocols were approved by the ethical committee of the Muséum National d’Histoire Naturelle (MNHN, Comité Cuvier, Licence: 68–002) and by the “Direction départementale de la cohésion sociale et de la protection des populations” (DDCSPP) (No. C75-05-15). The experiments performed at the Institute for Medical Microbiology, Immunology and Parasitology (IMMIP) of the University Hospital Bonn were in accordance to the European Union animal welfare guidelines and all protocols were approved by the Landesamt für Natur, Umwelt und Verbraucherschutz, Cologne, Germany (AZ 84–02.04.2015.A507; 84–02.04.2012.A140).

The procedures used for the production of *O*. *volvulus* forest strain third-stage-larvae (L3) were approved by an NIH accredited Institutional Review Board of the Medical Research Council Kumba, Cameroon (Protocol 001) and by Le Comité National d’Ethique de la Recherche pour la Santé Humaine, Yaoundé, Cameroon (Protocol 677). The cryopreserved third-stage larvae (L3) were shipped to the New York Blood Center in liquid nitrogen and upon arrival in New York were stored in liquid nitrogen. All protocols using the L3 cryopreserved samples in this study were approved by the New York Blood Center's IRB (Protocol 321 and Protocol 603–09). All L3 samples were anonymized. The peripheral blood mononuclear cells (PBMCs) used to culture *O*. *volvulus* L3 were isolated from human leukopaks collected from healthy donors following the New York Blood Center’s approved IRB protocol (Protocol 420). The de-identified human leukopaks were obtained from the New York Blood Center Component Laboratory. The New York City Blood Center obtained written informed consent from all participants involved in the study. All protocols were conducted in accordance with National Institutes of Health guidelines for the care and use of human subjects.

### *In vivo* analysis using *L*. *sigmodontis*

The life cycle of the filarial nematode *L*. *sigmodontis* was maintained in the animal facilities at the MNHN and at the IMMIP. Host animals used for infections at the MNHN were BALB/c mice purchased from Envigo, France, and jirds bred in the animal facilities on a 12 h light/dark cycle. Host animals used for infections at the IMMIP were BALB/c mice and jirds obtained from Janvier (Saint-Berthevin, France). All animals were infected between 6–8 weeks of age. Only female animals were used in this study.

At MNHN, mice were inoculated with 40 L3 in 200 μl of RPMI 1640 by sc injection in the left lumbar area. Infective L3 were recovered from the vector, the tropical rat mite *Ornithonyssus bacoti*, as previously described [29]. At IMMIP, mice and jirds were naturally infected by overnight contact with infected *O*. *bacoti* mites [30].

Infected mice were treated *per os* with a commercial formulation of oxfendazole (Dolthene) or by sc injection twice daily (BID, *bis in die*–twice a day) or once a day (QD, *quaque die*–once a day). Jirds were treated by oral gavage with oxfendazole or by sc injection of flubendazole (Sigma-Aldrich, Germany). Treatments were initiated at 33–35 days post-infection (dpi) in mice and 12 weeks after infection in jirds for either 1, 5 or 10 consecutive days. Doses in mg of drug substance per kg of body weight of animals are indicated in the text. Exposure of mice to oxfendazole was confirmed by dry spot analysis that was taken 24 h after the oral gavage at day 1–7 and day 10 of treatment. Therefore, 8 μl of peripheral blood were taken from the *vena facialis* and directly added to protein saver cards (Whatman 903 Protein saver card, Sigma-Aldrich, Germany). The dry spot analysis was performed by WuXi AppTec Co.,Ltd (China).

Mice were sacrificed at 75–78 dpi. Jirds were sacrificed 16 weeks after the first administration of treatment. The kinetics of microfilaremia in jirds were followed weekly from 70 dpi until necropsy. Samples of 10 μl of blood were taken from the femoral vein of jirds.

The mice were anesthetized then sacrificed by bleeding (at MNHN) or by an overdose of isoflurane (at IMMIP). At the MNHN, the pleural cavity was washed 10 times with 1 ml of cold phosphate buffered saline (PBS) to collect filariae as previously described [29]. At the IMMIP, the pleural cavity was washed with 1 ml of PBS. In both laboratories the remaining worms were isolated using forceps. The recovered *L*. *sigmodontis* adult worms were counted and analyzed by light microscopy to identify males and females.

In jirds, the female *L*. *sigmodontis* adult worms isolated at 16 weeks after treatment initiation were analyzed for embryogenesis as previously described [31]. Briefly, single female adult worms were homogenized in 20 μl Hinkelmann solution (0.5% eosin Y, 0.5% phenol, 0.185% formaldehyde in aqua dest.) and 80 μl PBS, and the embryonal stages eggs, morulae, pretzel, and stretched microfilariae were enumerated.

To investigate the *in vivo* efficacy of oxfendazole on microfilariae, microfilariae were isolated from peripheral blood of *L*. *sigmodontis*-infected cotton rats according to Chandrashekar *et al*. (1984) using a sucrose percoll gradient [32]. 100,000 microfilariae were injected into the tail vein of naïve mice as previously described [33]. Microfilariae-injected mice were treated with a commercial formulation of oxfendazole (Dolthene) twice daily with 2 x 12.5 mg/kg *per os*. As a positive control, additional mice were treated with a single intraperitoneal dose of ivermectin (5 mg/kg) in DMSO/PEG400/H_2_0_dest_ with a ratio of 1:7:2 according to Halliday et al. [34]. Control animals received no treatment.

### *In vitro* drug screening using *O*. *gutturosa* adult worms

*O*. *gutturosa* adult male worms were obtained post mortem from freshly slaughtered cattle. The worms were dissected from the nuchal ligament connective tissues of naturally infected cattle in Gambia, West Africa; the International Trypanotolerance Centre, Banjul, purchased the material from local butchers for use in this study. The adult worms were transferred individually to each well of a sterile 24-well (2 ml) plate (Fisher Scientific, UK) and maintained for at least 24 h in culture before use. The medium used was Minimum Essential Medium (MEM) with Earl’s Salts and L-Glutamine (Life Technologies Ltd, UK) supplemented with 10% heat inactivated new born calf serum (Life Technologies Ltd, UK) and 200 units/ml penicillin, 200 μg/ml streptomycin and 0.5 μg/ml amphotericin B (Life Technologies Ltd, UK). Only normally active worms were used for the test and all assays were conducted at 37°C under an atmosphere of 5% CO_2_ in air. Oxfendazole, albendazole (Sigma-Aldrich, UK) and flubendazole (Janssen Pharmaceutica) were supplied as dry solids and the positive control drug used was Immiticide (melarsomine dihydrochloride, Merial, USA). Known amounts of these compounds were solubilized in 1 ml DMSO. All stock solutions were allowed to stand at room temperature prior to use. Oxfendazole, albendazole and flubendazole were screened using serial 1 in 4 drug dilutions (5.0·10^−5^ M, 1.25·10^−5^ M, 3.1·10^−6^ M and 7.8·10^−7^ M), with the positive control at concentrations of 1.25·10^−5^ M, 3.1·10^−6^ M, 7.8·10^−7^ M and 1.9·10^−7^ M. The benzimidazoles (4 worms/drug concentration) were compared to untreated controls (6 worms/group) and the positive control (2 worms/drug concentration). Drug efficacy was assessed by the measurement of mean worm motility scores on a scale of 0 (immotile) to 10 (maximum) every 24 h, terminating at 120 h, using an Olympus inverted microscope. The results are reported as a percentage of the maximum score obtainable (100%) by calculating the motility index scores. The test drug is considered active when a motility reduction of ≥50% is observed by comparison to the untreated controls. In addition, biochemical evaluation of worm viability was assessed using MTT/formazan colorimetry. The MTT assay was carried out after the last motility reading (120 h). Single intact worms were placed in each well of a 48-well plate (Fisher Scientific, UK) containing 0.5 ml of 0.5 mg/ml MTT (Sigma, UK) in PBS solution, and then incubated for 30 min at 37°C. The worms were removed, blotted carefully, and individually transferred to separate wells of a 96-well microtitre plate, each containing 200 μl of DMSO to solubilize the formazan. After 1 h the plate was gently agitated to disperse the color evenly and the absorbance value (optical density, OD) of the resulting formazan solution was determined at 490 nm using an absorbance microplate reader (Biotek ELx800, Fisher Scientific, UK). The results are expressed as the mean OD per drug concentration. Inhibition of formazan formation is correlated with worm damage or death.

### *In vitro* drug screening using *O*. *volvulus* pre-adult worms

Cryopreserved *O*. *volvulus* L3 were thawed, washed and co-cultured with normal human PBMC as previously described [35, 36]. Larvae were cultured at 37°C with 5% CO_2_ until day 6 when the molting was observed. The L4 larvae were then transferred into a transwell (Sigma #CLS3472), ~10 L4 per transwell within a 24-well plate and over a monolayer comprised of Human Umbilical Vein Endothelial Cells (HUVEC; Lonza Inc., Allendale, NJ) seeded at a density of 1 x 10^4^ cells per well. The larvae were cultured for 80 days using L4 complete media: 1:1 NCTC-109: MEM-alpha and EBM-2 (Clonetics EBM-2 Lonza# CC-3156) at a 60:40 ratio. The media was supplemented with 25% heat-inactivated FBS, 1 x Antibiotic-Antimycotic, 1% Glucose (Sigma #G8769), 1% Sodium Pyruvate and 1% ITS (Life Technologies #11360–070 and #51500–056), 0.1% Lipid Mixture-1 (Sigma # L0288), and 1% Non-Essential Amino Acids (Life Technologies# 11140–050). Medium was exchanged 3 times a week and fresh HUVEC monolayers were prepared weekly. A week prior to performing the drug screening assay, the L4 day 80 that has molted to pre-adult worms, L5, were transferred from the transwells into a small petri dish containing fresh complete media. 7–10 L5 worms were retrieved from the petri dish and placed in new transwells containing a freshly prepared HUVEC monolayer. Oxfendazole (Sigma) was dissolved in DMSO and added to the L5 larvae using serial 2 x concentrations of the drug to achieve final concentrations of 1·10^−5^, 3·10^−6^, 1·10^−6^, 3·10^−7^, 1·10^−7^ M, and 3.3·10^−8^ M. Plates were incubated at 37°C and 5% CO_2_ for 14 days during which 0.5 ml of medium was replaced with fresh medium containing the drug every 2–3 days. Negative control worms were placed in complete media with 0.05% DMSO. After 14 days of treatment, the drug containing media was replaced by complete media without drug. Continuing this assay for a further 19 days, the worms were supplied with fresh media every 2–3 days and the weekly use of fresh HUVEC monolayers. Worm motility was assessed every 2–3 days over the entire assay period using the following scores: 100% motility, constant coiling movement; 75% motility, slower coiling; 50% motility, slow and intermittent movement; 25% motility, very slow movement or twitching; and 0% motility, no movement. All drug concentrations were tested in duplicate. The percent inhibition of motility for treated L5 worms were calculated with respect to the movement of L5 in wells containing DMSO in normal complete media (negative control). IC_50_ values were calculated using Graph Pad Prism v8.3.

### *In vitro* drug screening using *O*. *lienalis* microfilariae

A single large batch of *O*. *lienalis* microfilariae was obtained post-mortem from the peri-umbilical skin area of freshly slaughtered, naturally infected cattle in the UK following the procedure described by Townson and Tagboto [37] and the references therein. The extracted microfilariae were cryopreserved using a 2-step incubation technique with ethanediol as a cryoprotectant, stored in liquid nitrogen and thawed when required for immediate use. For the *O*. *lienalis* 5-day motility assay, a confluent layer of a mammalian cell line of monkey kidney (LLCMK2, ECACC, UK) was used as feeder cells and maintained in Minimum Essential Medium with Earl’s Salts and L-Glutamine (Life Technologies Ltd, UK) supplemented with 10% heat inactivated new born calf serum (Life Technologies Ltd, UK) and antibiotics/antimycotics (200 units/ml penicillin, 200 μg/ml streptomycin and 0.5 μg/ml amphotericin B; Life Technologies Ltd, UK). These assays were performed using sterile 96-well (200 μl) plates (Fisher Scientific, UK) and conducted at 37°C under an atmosphere of 5% CO_2_ in air. Using a dissecting microscope and micropipette, microfilariae were then transferred in groups of 5 into each well of a 96-well plate. Oxfendazole, albendazole (Sigma Aldrich, UK) and flubendazole (Janssen Pharmaceutica) were supplied as dry solids and the positive control drug used was Immiticide (melarsomine dihydrochloride, Merial). Known amounts of these compounds were solubilized in 1 ml DMSO. All stock solutions were allowed to stand at room temperature prior to use. Oxfendazole, albendazole, flubendazole and the positive control Immiticide (5 microfilariae in 1 well/drug concentration), were screened in duplicate using serial 1 in 4 drug dilutions (oxfendazole, albendazole, flubendazole: 5.0·10^−5^ M, 1.25·10^−5^ M, 3.1·10^−6^ M and 7.8·10^−7^ M; Immiticide: 1.25·10^−5^ M, 3.1·10^−6^ M, 7.8·10^−7^ M and 1.9·10^−7^ M) with untreated controls (5 microfilariae in each of 6 wells). After 120 h of drug exposure, the motility of the microfilariae was classified using an Olympus inverted microscope as normal (continuous rapid sinuous movement, scored as 3), marginally impaired (slower than normal movement, scored as 2), severely impaired (scored as 1), or immotile (scored as 0). The results were then calculated as motility index scores, percentages of the maximum scores obtainable (100).

### Pharmacokinetic analyses

Female BALB/c mice (6 to 9-weeks old) were used to determine the plasma concentrations of oxfendazole. Animals were allocated to groups of three animals per dose, such that the mean weight of animals in each group was similar. All animals had access to a certified rodent diet (Beijing Keao Xieli Feed Co, Ltd) and water *ad libitum*. In this study, all animals were fasted 12 h before dosing and returned to food 4 h post dose.

Commercially available oxfendazole (Sigma Aldrich and Dolthene, Merial) was administered to animals. A suspension of oxfendazole in 0.1% Tween 80 and 0.5% hydroxy-ethyl-cellulose was administered by the parenteral route. A commercially available formulation of oxfendazole, Dolthene, was diluted in corn oil and administered by oral gavage.

After administration of the drug substances by oral gavage, blood samples were collected from each animal via the saphenous or mandibular veins at 0.25, 0.5, 1, 2, 4, 8, 12 and 24 h post dosing. For administration via the sc route, samples were collected from each animal 1, 3, 6, 9, 12, 18, 24, 36, 48, 72, 96, and 120 h post dosing. All samples were transferred into plastic tubes containing K2-EDTA (0.5 M) as an anti-coagulant and stored on ice until plasma preparation by centrifugation at 5°C (3000 g, 15 min).

Analysis was conducted by LC-MS/MS (API 4000). Mobile phases were mixtures of acetonitrile (ACN) and water to which formic acid and 1 mM NH_4_OAc were added (0.025%, v/v). The ratio ACN/water was 5:95 (v/v) in the mobile phase A and 95:5 (v/v) in the mobile phase B. For oxfendazole, using an ACQUITY UPLC BEH C18 (2.1×50 mm, 1.7 μm) column a linear gradient was pumped at a flow rate of 0.6 ml/min, changing from a 90% mobile phase A, to a 45% mobile phase A at 1.2 min, to a 10% mobile phase A from 1.4 min to 1.7 min.

Electrospray positive ionization with selected ion MS/MS monitoring was used for the determination of oxfendazole: m/z 316.0/159.1.

### Statistical analysis

Statistical analyses were performed using GraphPad Prism Software Version 8.2.1 (San Diego, USA). The Shapiro-Wilk normality test was performed, and statistical significance was tested between treatment groups and the control group using the Kruskal-Wallis test followed by Dunn’s multiple comparison test or two-way ANOVA followed by Dunnett’s multiple comparison test. P-values of <0.05 were considered statistically significant.

## Results

### Oxfendazole has a moderate effect on *O*. *gutturosa* and *O*. *volvulus* adult worm motility *in vitro*

**Fig 1:**
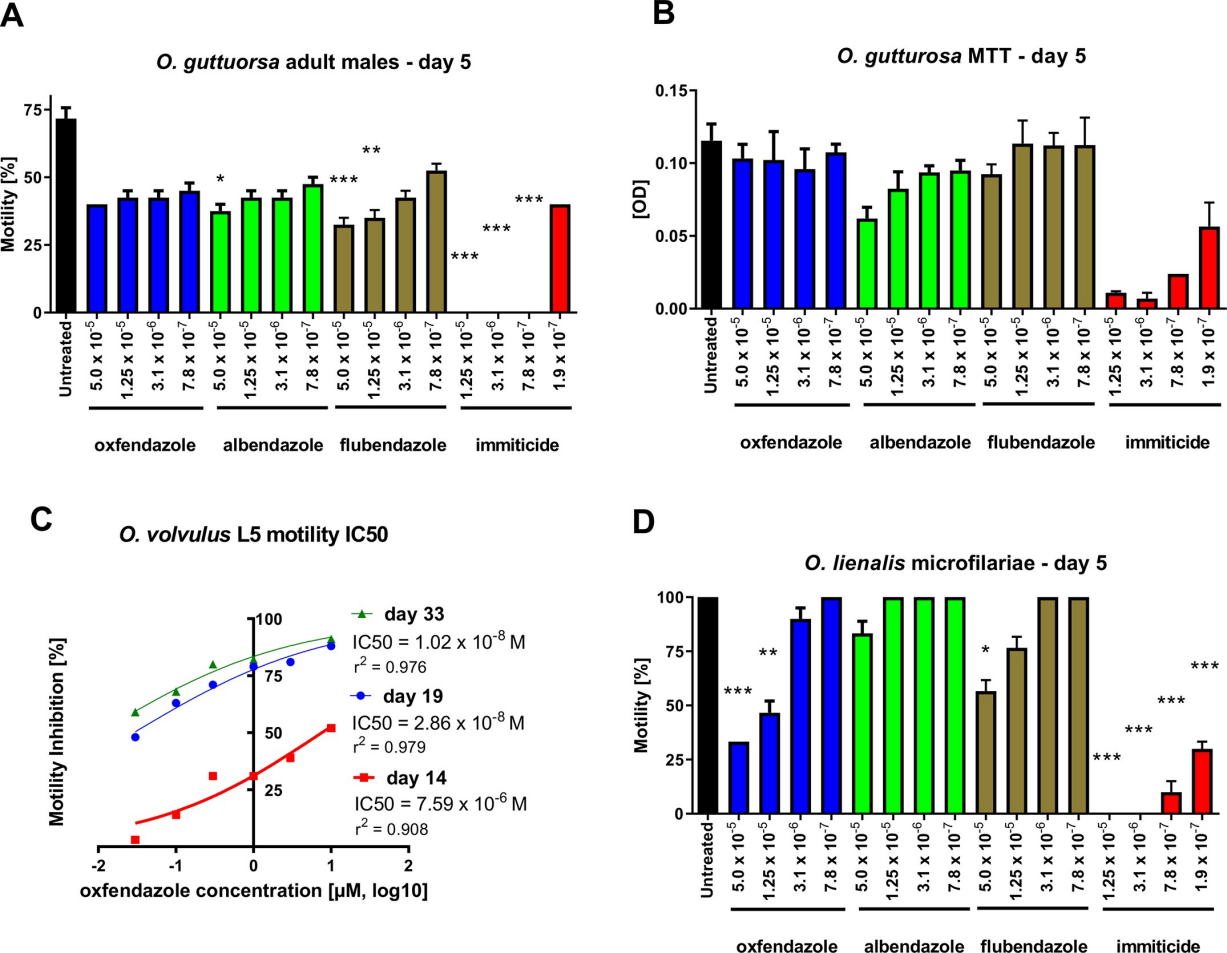
Oxfendazole has a moderate effect on *O*. *gutturosa* and *O*. *volvulus* adult worm motility *in vitro*. *Onchocerca* parasites were exposed to oxfendazole, albendazole, flubendazole and Immiticide (melarsomine used as a positive control) at the final concentrations indicated. (A) *O*. *gutturosa* adult male motility at 120 h. (B) *O*. *gutturosa* adult male MTT assay represented as mean optical density (OD) values at 120 h. For (A) and (B), means + SEM from 4 individual adult worms per drug concentration and 6 untreated adult worms are shown. For Immiticide, 2 adult worms per concentration were used. Vertical lines represent the standard deviation. (C) *O*. *volvulus* pre-adult L5 worms were cultured with oxfendazole over 14 days followed by 19 days in complete media without drug. Shown is the IC50 of the motility inhibition at 14 (red square), 19 (blue circle) and 33 days (green triangle) of culture. Each condition was assayed with 7–10 worms total divided in two replicate wells. (D) *O*. *lienalis* microfilariae motility at 120 h. Means + SEM from 10 individual worms per drug concentration and 10 untreated worms are shown. Vertical lines represent the standard deviation. (A) Kruskal-Wallis H-test: χ^2^ = 49.0, *df* = 17, p<0.0001 followed by Dunn’s multiple comparison test; (B) Kruskal-Wallis H-test: χ^2^ = 32.15, *df* = 17, p<0.0096 followed by Dunn’s multiple comparison test; (D) Kruskal-Wallis H-test: χ^2^ = 156.1, *df* = 16, p<0.0001 followed by Dunn’s multiple comparison test; *p<0.05; **p<0.01; ***p<0.001.

The activity of oxfendazole was assessed *in vitro* against male *O*. *gutturosa* adult worms in a 5-day motility and MTT (3-[4,5-dimethylthiazol-2-yl]-2,5-diphenyltetrazolium bromide) assay by testing a range of concentrations (5.0·10^−5^ M to 7.8·10^−7^ M, 1 in 4 serial dilutions); the motility scores were measured over 120 h at 24 h intervals. Overall, compared to untreated controls, oxfendazole displayed moderate activity against adult male worms at the highest concentration of 5·10^−5^ M, with a motility reduction of 16% after 24 h, which decreased further to 44% after 120 h of drug exposure (Fig 1A; S2 Table). Similarly, the benzimidazoles flubendazole and albendazole showed moderate activities against *O*. *gutturosa* adult worms that was comparable to oxfendazole (Fig 1A; S2 Table). As expected, the positive control Immiticide (melarsomine dihydrochloride), a marketed treatment for *Dirofilaria immitis* (heartworm) infection in dogs, was more active; at 1.25·10^−5^ M and 3.1·10^−6^ M, parasites were completely immotile after 24 h. At a lower concentration of 7.8·10^−7^ M a complete motility inhibition was observed after 96 h. The effects of oxfendazole, albendazole, flubendazole and Immiticide with regard to the viability of the worms were also reflected by MTT colorimetry: formazan formation was inhibited by only 11% using 5·10^−5^ M oxfendazole and by 79 to 94% using 7.8·10^−7^ M to 1.25·10^−5^ M Immiticide (Fig 1B; S2 Table).

In addition, activity of oxfendazole was tested *in vitro* against *O*. *volvulus* pre-adult (L5 stage) worms by exposing the worms for 14 days to oxfendazole, followed by 19 days of culture with no drug. Whereas control groups exhibited consistent motility values over the complete observation period of 33 days, oxfendazole treatment caused a dose- and time-dependent reduction of the motility of the *O*. *volvulus* L5 worms. After *in vitro* drug exposure for 14 days, the IC50 of motility inhibition by oxfendazole was 7.59·10^−6^ M (Fig 1C). Subsequent washout of the drug did not restore the motility of the L5 worms, but resulted in an almost complete inhibition of motility after 19 and 33 days of culture (5 and 19 days without drug exposure) with IC50s of 2.86·10^−8^ and 1.02·10^−8^ M, respectively.

*O*. *lienalis* microfilariae motility decreased at the highest evaluated concentrations of oxfendazole, 5·10^−5^ M and 1.25·10^−5^ M by 67% and 53% respectively. No effect was observed at lower concentrations and albendazole and flubendazole were even less active against the microfilariae (Fig 1D). In contrast, Immiticide completely inhibited microfilariae motility at concentrations equal or above 3.1·10^−6^ M (Fig 1D). Thus, the oxfendazole-mediated inhibition of motility against *O*. *lienalis* microfilariae is modest *in vitro*.

### Oxfendazole mediates a dose-dependent macrofilaricidal efficacy *in vivo*

**Fig 2:**
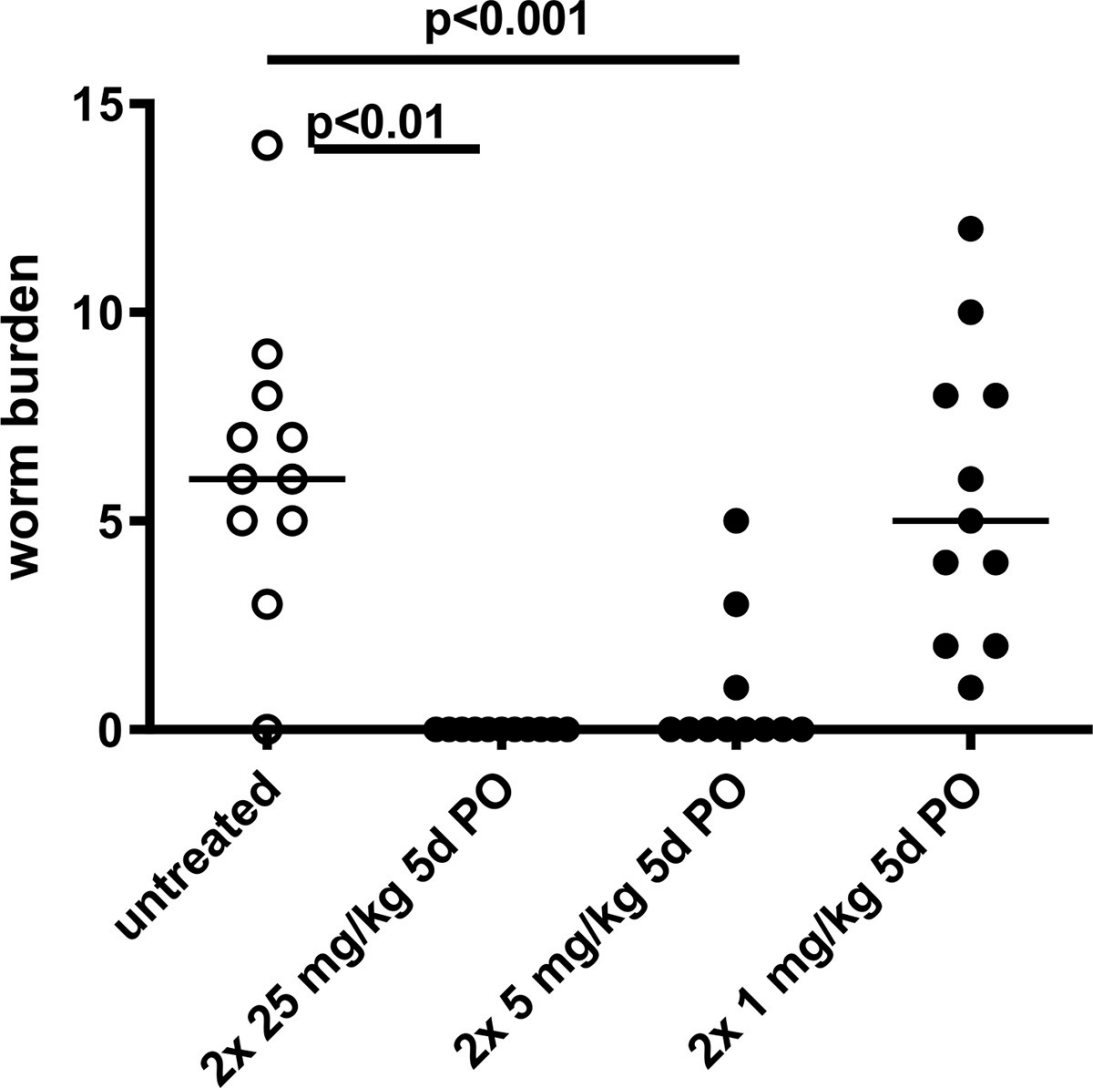
Oxfendazole provides sterile cure in a dose-dependent manner. *L*. *sigmodontis* adult worm burden of mice 75 dpi, subcutaneously infected with 40 infective L3 larvae and treated orally (PO) twice per day for five consecutive days (5d) starting at 35 dpi with 1, 5, or 25 mg/kg body weight of oxfendazole (n = 11 per group). Untreated controls received no oral treatments. Medians of a single experiment are shown. Kruskal-Wallis H-test: χ^2^ = 29.62, *df* = 3, p<0.0001 followed by Dunn’s multiple comparison test.

Macrofilaricidal efficacy of oxfendazole was investigated *in vivo* in mice infected with *L*. *sigmodontis* 35-days post infection (dpi), when worms have already molted to their adult stage. Treatment was administered by oral gavage for 5 days with two daily (BID) doses of 2 x 1, 2 x 5 or 2 x 25 mg/kg. While untreated controls had a median of 6 adult worms (range 0–14) at necropsy (75 dpi), sterile cure was achieved in all animals receiving BID treatments of 2 x 25 mg/kg oxfendazole (p<0.01, 100% reduction, Fig 2). Treatments with the lower doses of 2 x 5 mg/kg and 2 x 1 mg/kg oxfendazole resulted in medians of 0 (p<0.001, 100% reduction, range 0–5 worms) and 5 (p>0.05, 16.7% reduction, range 1–12 worms) adult worms, respectively. These results show that an oral treatment of oxfendazole for 5 days reduces the *L*. *sigmodontis* adult worm burden in a dose-dependent manner and with doses as low as 2 x 5 mg/kg.

### Comparison of the pharmacokinetic profile by oral and subcutaneous administration

**Fig 3:**
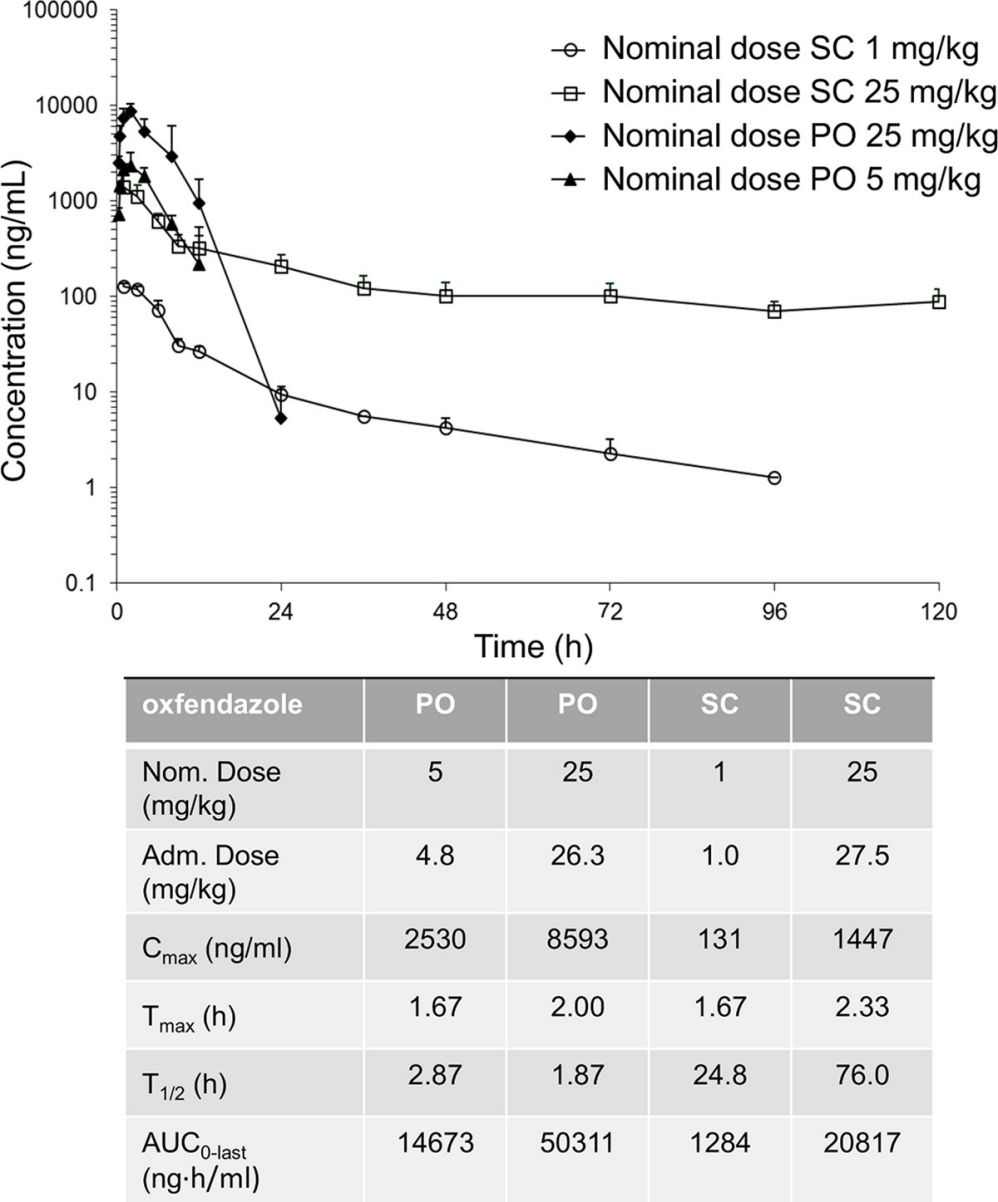
Pharmacokinetic profile of oxfendazole in mice following oral and subcutaneous administration. Maximum plasma concentrations (C_max_), time to C_max_ (T_max_), half-life (T_1/2_) and area under the curve at the last measured plasma concentration (AUC_0-last_) of oxfendazole in mice after oral (PO) or subcutaneous (SC) administration (n = 3 per group) at the indicated doses.

The plasma concentration of oxfendazole following oral or sc administration was determined in mice, in four satellite groups. Doses of 5 and 25 mg/kg were administrated orally and doses of 1 and 25 mg/kg were administered subcutaneously (Fig 3). Comparing oral and sc administration at 25 mg/kg, maximum plasma concentration (C_max_) is about 6 times higher in the oral treated group. Time to C_max_ (T_max_) occurs however in all groups at approximately the same time (T_max_ is approximatively 2 h). This data suggests a rapid absorption of oxfendazole via both routes of administration and a similar decline during the first 12 h (i.e. half-life T_1/2_ is approximatively 2 h). However, when administered subcutaneously, elimination of oxfendazole is biphasic with a terminal half-life (terminal phase starting 12 h after dosing) being approximatively 40 times longer compared to the T_1/2_ by oral route. Despite this, the area under the curve (AUC_0-last_) is 2.4 times higher when oxfendazole is administered orally compared to sc administration. Following oral administration of 5 and 25 mg/kg of oxfendazole a C_max_ of 8 and 27 μM, respectively, was reached. Of note, the metabolites of oxfendazole, fenbendazole and fenbendazole sulfone, are active against adult *O*. *gutturosa*, however they are less potent compared to oxfendazole.

### Maintenance of the minimal efficacious concentration of oxfendazole determines macrofilaricidal efficacy

To determine the minimal efficacious dose of oxfendazole, dose-response experiments were conducted in *L*. *sigmodontis* infected mice. Oxfendazole was administered BID *per os* (2 x 2.5, 2 x 12.5 mg/kg) or QD by sc injection (1, 5, 25 mg/kg) for five consecutive days. Treatments were initiated 35 dpi and necropsies were performed 78 dpi.

**Fig 4:**
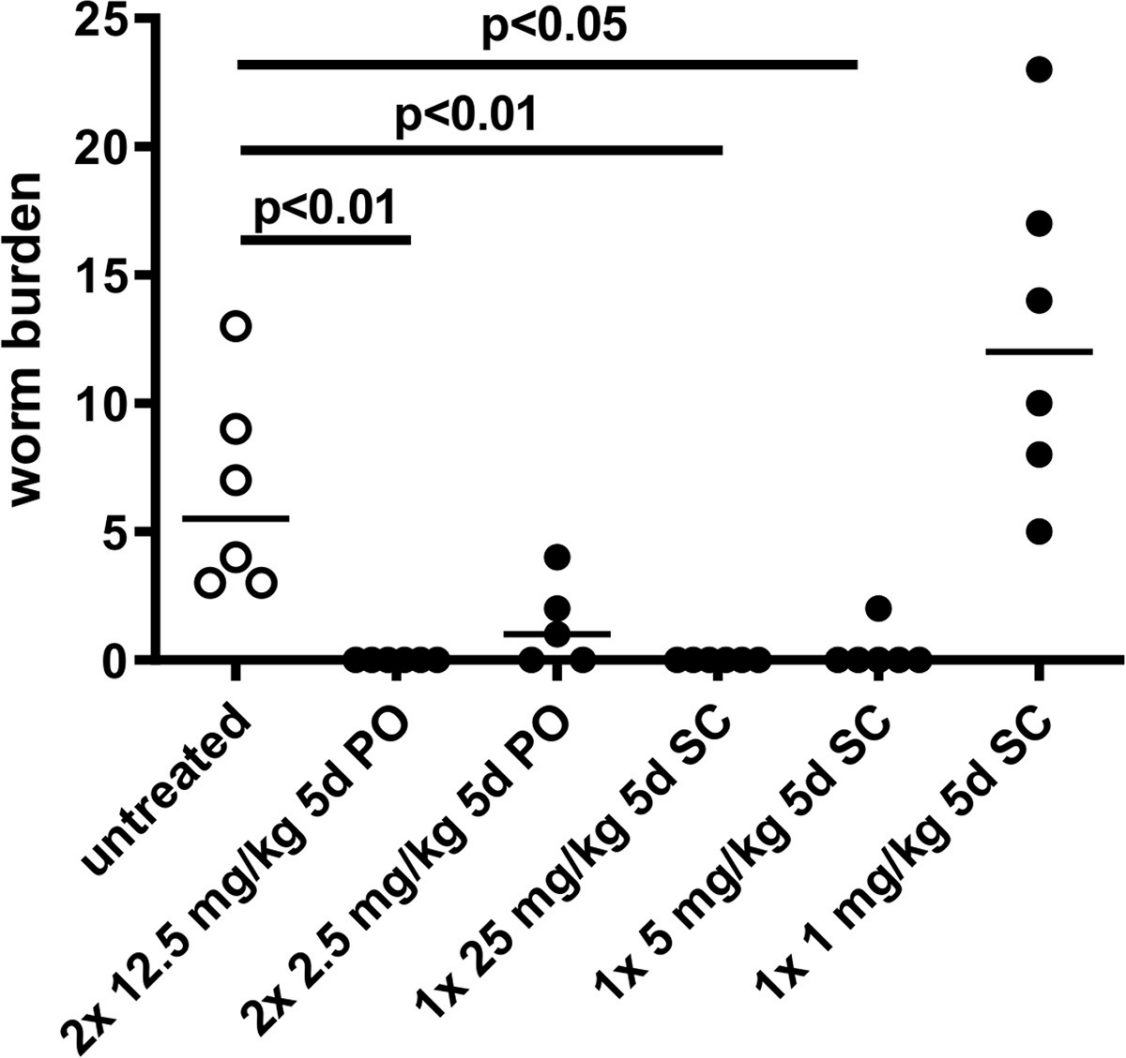
Comparison of the macrofilaricidal efficacy of oral and subcutaneous treatment routes for oxfendazole. *L*. *sigmodontis* adult worm burden 78 dpi in mice naturally infected with *L*. *sigmodontis* and treated for five consecutive days (5d) with oxfendazole twice per day orally (PO) with 2.5 (n = 5) or 12.5 mg/kg body weight (n = 6) and once per day subcutaneously (SC) (n = 6 per group) with 1, 5 or 25 mg/kg oxfendazole. Untreated controls (n = 6) received no oral or subcutaneous treatments. Medians of a single experiment are shown. Kruskal-Wallis H-test: χ^2^ = 29.26, *df* = 5, p<0.0001 followed by Dunn’s multiple comparison test.

Orally, sterile cure was achieved (100% adult worm reduction) at 2 x 12.5 mg/kg for 5 days (Fig 4) and at 2 x 25 mg/kg for 5 days (Fig 2). Sterile cure was achieved also when oxfendazole was administered by the sc route at the high dosing regimen (25 mg/kg QD for 5 days, Fig 4). At the mid sc dosing regimen (5 mg/kg QD for 5 days) sterile cure was achieved in all mice except one, for which two adult worms were recovered at necropsy. At the low sc dosing regimen (1 mg/kg QD for 5 days) no significant reduction of the worm burden was observed (Fig 4).

**Fig 5:**
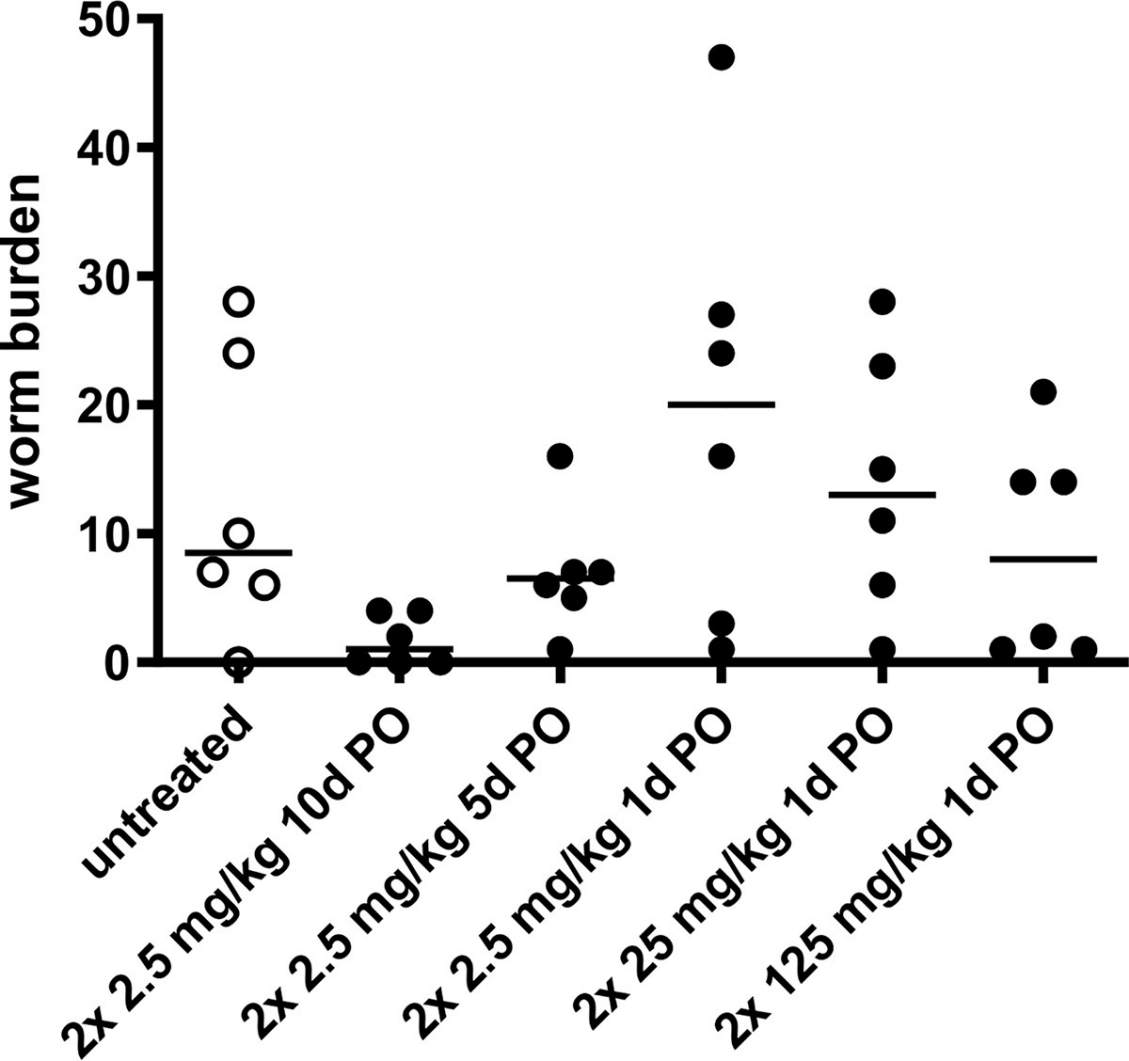
Extended oxfendazole treatment improves *L*. *sigmodontis* adult worm clearance. *L*. *sigmodontis* adult worm burden 75 dpi in mice naturally infected with *L*. *sigmodontis* and treated for one, five or ten consecutive days with oxfendazole starting at 33 dpi at indicated doses. Untreated controls received no oral treatments. Medians of n = 6 per group of a single experiment are shown. Kruskal-Wallis H-test: χ^2^ = 10.15, *df* = 5, p = 0.071.

Single oral BID dosing regimens were evaluated in mice with treatment start at 33 dpi. An increase of doses up to 125 mg/kg did not lead to a significant reduction of the adult worm burden compared to untreated controls at 75 dpi (Fig 5). In contrast, extended treatment duration improved efficacy when an oral treatment of 2 x 2.5 mg/kg is administrated for 1, 5 and 10 consecutive days. Reduction of the adult worm burden in comparison to the untreated control group, increased with extended treatment duration from 0 (median 20 worms, range 1–47 worms) to 23.5% (median 6.5 worms, range 1–16 worms) and 87.2% (median 1 worm, range 0–4 worms), respectively (Fig 5). These results are consistent with a time-dependent pharmacodynamic effect. A BID oral dosing regimen of 2 x 12.5 mg/kg for 5 days or 2 x 5.0 mg/kg for 10 consecutive days produced a macrofilaricidal efficacy above 85%.

### Oxfendazole treatment during patent *L*. *sigmodontis* infection provides macrofilaricidal efficacy and inhibits embryogenesis

Efficacy of oxfendazole against adult worms and microfilariae was evaluated in a chronic infection with *L*. *sigmodontis* in jirds. A BID oral treatment of 2 x 12.5 and 2 x 5 mg/kg for 10 consecutive days was administered to patent, microfilariae-positive animals. As macrofilaricidal comparator a positive control group was treated with 5 QD sc doses of 2 mg/kg flubendazole.

**Fig 6:**
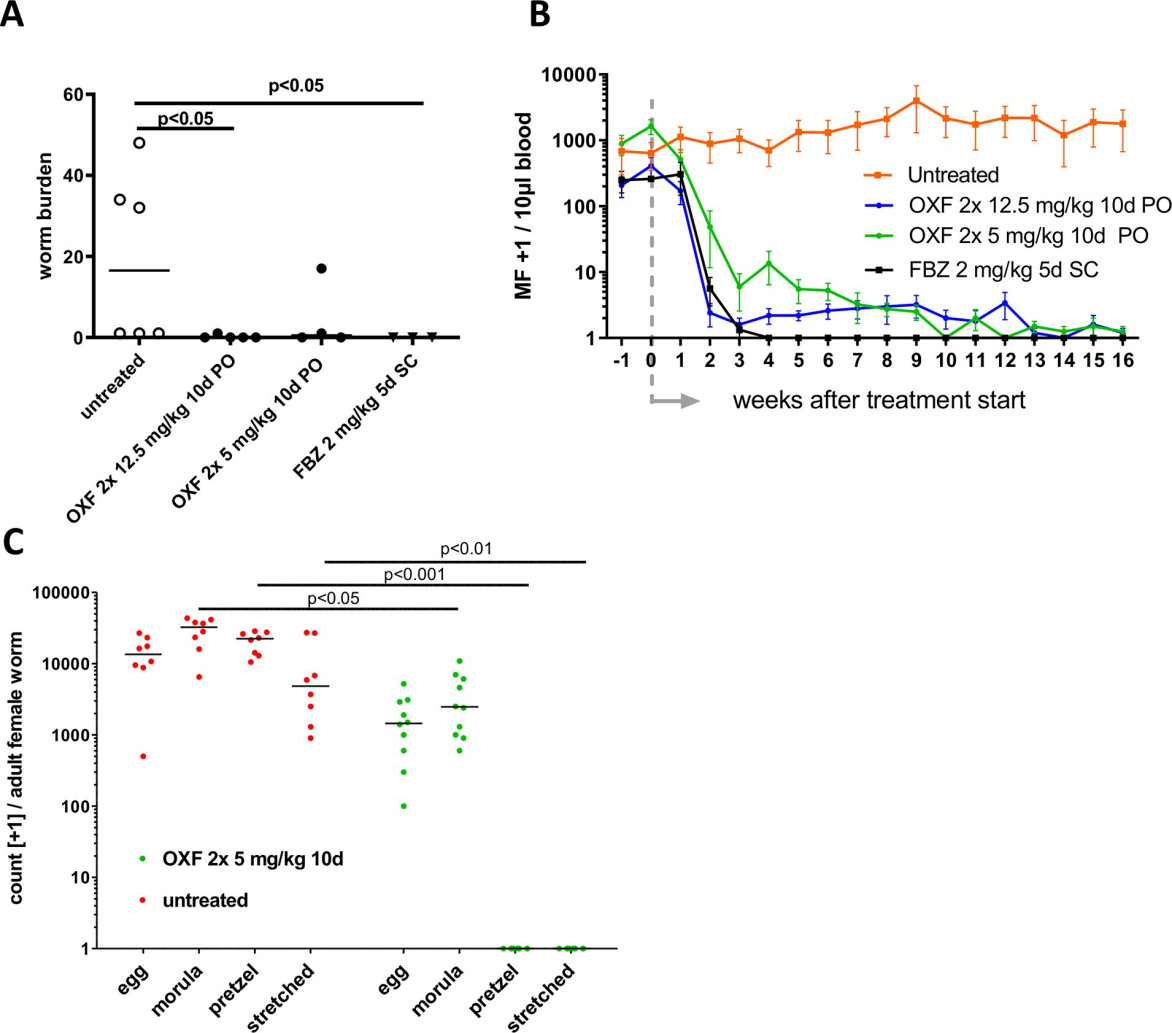
Oxfendazole treatment during patent *L*. *sigmodontis* infection demonstrates macrofilaricidal efficacy and inhibits embryogenesis in jirds. Microfilariae-positive jirds were treated 12 weeks post-infection (wpi) twice daily orally (PO) with 5 (n = 4) or 12.5 mg/kg (n = 5) oxfendazole (OXF) for a total of 10 consecutive days or subcutaneously with 2 mg/kg flubendazole (FBZ; n = 3) for 5 consecutive days. Untreated animals (n = 6) served as negative controls and necropsies were performed on all animals 16 weeks post-treatment (wpt). (**A**) *L*. *sigmodontis* adult worm burden at necropsy and (**B**) peripheral blood microfilariae levels over time. (**C**) Embryograms of female adult worms isolated from untreated (n = 8) and oxfendazole (n = 10) treated jirds (10 days, BID, 5 mg/kg) showing the total number of eggs, morulae, pretzel and stretched microfilariae per female worm. (**A, C**) Medians of one representative of two independent experiments are shown. (**B**) Means ± SEM of one representative of two independent are shown. (**A**) Kruskal-Wallis H-test: χ^2^ = 10.62, *df* = 3, p = 0.004 followed by Dunn’s post-hoc test. (**B**) Two-way ANOVA: *F*_(3, 252)_ = 24.29, p<0.0001 followed by Dunnett’s multiple comparison test. p<0.0001 for all treatment groups in comparison to untreated controls. (**C**) Kruskal-Wallis H-test: χ^2^ = 60.16, *df* = 7, p<0.0001 followed by Dunn’s multiple comparison test.

Significant reduction in adult worm burden (p<0.05) was obtained at 16 weeks after treatment start using the high dosing regimen of oxfendazole (Fig 6A), which provided complete clearance of adult worms in four animals out of five. In the low dosing regimen complete clearance of adult worms was achieved in two animals out of four.

In the flubendazole group all microfilariae were cleared from peripheral blood at week 4 after treatment start (Fig 6B) and no adult worms were recovered at necropsy (Fig 6A). Similarly, 3 weeks after treatment, more than 99% of microfilariae were cleared from the peripheral blood in both oxfendazole groups (Fig 6B). Between weeks 14 and 16 after treatment, few microfilariae were observed in the peripheral blood in one jird out of five and in one jird out of four in the high and low dose oxfendazole group, respectively. Embryograms performed on alive recovered female worms from the low dose group revealed that embryogenesis was inhibited, as only early embryonic stages (egg, morulae) were present and later embryonic stages (pretzel, microfilariae) were completely absent (Fig 6C). Oxfendazole is therefore highly effective in the patent *L*. *sigmodontis* filarial model, eliminating adult worms, inhibiting embryogenesis and production of microfilariae, which leads to the clearance of microfilariae from the peripheral blood.

### Oxfendazole has no efficacy against *L*. *sigmodontis* microfilariae *in vivo*

**Fig 7:**
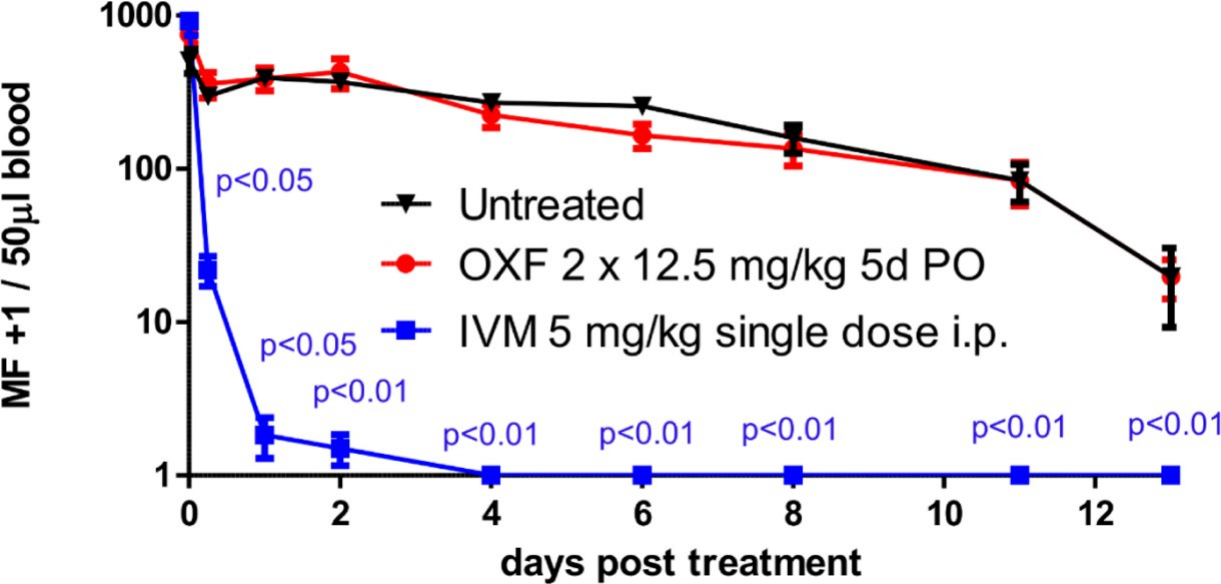
Oxfendazole has no *in vivo* efficacy against microfilariae of *L*. *sigmodontis*. Time-course of microfilariae counts in 50 μl peripheral blood of mice injected with 100,000 microfilariae and treated the following day with oxfendazole twice daily (OXF, BID) for five days with 12.5 mg/kg *per os* or a single intraperitoneal dose of ivermectin (IVM, 5 mg/kg). Control animals received no treatment. Means ± SEM of 6 mice per group of one single experiment are shown. Two-way ANOVA: *F*_(2, 135)_ = 29.16, p<0.0001 followed by Dunnett’s multiple comparison test. p<0.0001 for IVM and p = 0.60 for oxfendazole in comparison to untreated controls.

Ivermectin and DEC, clear microfilariae from patient’s skin and eventually cause pathology such as the DEC-mediated Mazzotti reaction in onchocerciasis patients [1, 2]. We have therefore investigated the direct effect of oxfendazole on *L*. *sigmodontis* microfilariae *in vivo*. Naïve mice injected with *L*. *sigmodontis* microfilariae were treated with a BID dose of 2 x 12.5 mg/kg of oxfendazole for 5 consecutive days. In comparison with the untreated control group the response was similar with a marginal clearance of microfilariae over time (Fig 7). In contrast, mice treated with a single intraperitoneal injection of ivermectin (5 mg/kg) had a statistically significant reduction of microfilaremia by 6 h after treatment. From day four after treatment, all ivermectin-treated animals were microfilariae negative until the end of the experiment. These results demonstrate that oxfendazole has *in vivo* no direct microfilaricidal effect in the *L*. *sigmodontis* model. If oxfendazole has a comparable life-cycle stage-specific activity in human filarial infection, oxfendazole could be a selective macrofilaricide with the potential to prevent the Mazzotti reaction and to be used for treatment of patients co-infected with onchocerciasis and loiasis.

### Prediction of human pharmacokinetics and efficacious human dose of oxfendazole

Several independent methods have been applied to predict key pharmacokinetic parameters for oxfendazole in humans. Table 1 provides brief summaries and the underlying literature for all methods, including final predicted values determined by considering several published approaches (for all formulas used see references [38–40]). Oxfendazole is predicted to be a low clearance (CL) drug in humans (~2% liver blood flow). Two independent methods that considered species differences in plasma protein binding (PPB), namely *in vivo* allometric scaling and scaling of *in vitro* hepatocyte intrinsic clearance, resulted in an identical prediction of oxfendazole total human plasma clearance of ~0.4 mL/min/kg. In contrast, direct allometry (i.e. not considering PPB) resulted in a much higher predicted clearance. The latter result was judged unrealistic since not correcting for PPB resulted in an allometric β exponent of ~1.2, which is far above the theoretical β value of ~0.75, while allometry considering PPB resulted in a quasi-ideal β of ~0.74. Further, oxfendazole is predicted to have a low-to-moderate volume of distribution at steady state (V_ss_ ~0.5 L/kg as the mean of all independent calculations), which is approximately the volume of body water, indicating that the compound distributes out of the blood compartment. The resulting half-life of oxfendazole has been estimated via the V_ss_/CL ratio as well as via the direct correlation with the half-life obtained in pre-clinical species (see Table 1 for formulas). The latter may represent a terminal half-life due to the formulas used, while the former is considered to represent the half-life under steady state conditions. Since prediction via the V_ss_/CL ratio did not result in a shorter half-life, the mean of all approaches was used for a final half-life prediction at steady state of ~10 h. Finally, preclinical bioavailability data indicate that the development of a human formulation will likely not pose a challenge for the following reasons. First, the predicted clearance in humans of 0.4 mL/min/kg is very small and equivalent to ~2% of liver blood flow. In such cases, current predictive software (e.g. GastroPlus) set the extraction ratio (ER; which is representing mathematical reduction of bioavailability due to intestinal and liver first pass metabolism) at ER<0.1. In practical terms this means that there is virtually no negative impact on bioavailability due to first pass metabolism. Secondly, as a simple formulation of oxfendazole is already used successfully in various animal species and bioavailability in rat, sheep and cattle of >50% was reported [37], indicating that in several species (larger and smaller than humans from an allometric standpoint) trans-intestinal absorption as well as low first pass metabolism could be demonstrated. In summary, the current data indicate that a human formulation achieving >50% bioavailability is achievable. Despite this, a conservative estimate for oral bioavailability of 30% was used for the prediction of the efficacious dose in humans.

**Table 1:** Human pharmacokinetic prediction for oxfendazole.

Human PK Prediction for Oxfendazole^a^		
**Clearance (CL)**	**(mL/min/kg)**	**Comments**^**b**^
rat-dog allometry	8.0	CL = α x BW^β^
rat-dog allometry (+PPB^c^)	0.4	unbound CL = α x BW^β^
hepatocyte CL scaling	0.4	well-stirred model using fu x CL_int_
**Final**	**0.4**	
**Vol. of Distribution (V**_**ss**_**)**	**(L/kg)**	**Comments**^**b**^
rat-dog allometry	1.2	V_ss_ = α x BW^β^
via rat (with PPB^c^)	0.2	V_ss,h_ = mean fu,h x (V_ss,y_/fu,y)
via dog (with PPB^c^)	0.1	
**Final**	**0.5**	
**Half-Life (HL)**	**(h)**	** Comments**
via predicted Vss/CL	14.4	T_1/2_ = ln_2_ x (V_ss_/CL)
rat-hum correlation	12.1	log(T_1/2_ human) = 0.906 log (T_1/2_ rat) + 0.723
dog-hum correlation	4.2	log(T_1/2_ human) = 0.934 log (T_1/2_ dog) + 0.433
**Final**	**10.2**	
**Bioavailability (F)**	**(%)**	** Comments**
rat/sheep/cattle	>50	published data [41]
rat	~35	Published data [42]
dog	~10	in-house data at high dose of 25 mg/kg
**Final**	**30**	

^a^Formulas and underlying theories to predict CL, Vss, HL see [38] and for F see [40]

^b^allometric formula to correlate CL or Vss with body weight (BW) via power function see [38]

^c^plasma protein binding

The predicted human pharmacokinetic parameters for oxfendazole were used to estimate the efficacious human dose following several days of administration (steady state conditions). The target concentrations to achieve efficacy in humans were deducted from the average steady state (C_ave,ss_) and minimal steady state concentrations (C_min,ss_) needed for efficacy in the *L*. *sigmodontis* mouse and jird models (for mouse with and without consideration of differences in plasma protein binding compared to humans). The resulting daily doses needed to reach all assumed target concentrations for these scenarios in humans were calculated to be between 1.5 and 4.1 mg/kg (average all methods: 2.7 mg/kg assuming a 70 kg subject). Considering that daily doses of more than 10-fold higher (up to 60 mg/kg) were administered to healthy volunteers with acceptable side effects [20], and a multiple ascending dose study with oxfendazole treatments for five consecutive days with 3, 7.5 and 15 mg/kg were successfully completed (https://clinicaltrials.gov/ct2/show/NCT03035760), the dose modeling indicates that a therapeutic dose in humans can be established including a safety margin. Exposure in humans at the predicted efficacious dose (i.e. 2.7 mg/kg) appears to be in good agreement with a dosing regimen providing sterile cure in the filarial animal models and lacking a microfilaricidal efficacy.

## Discussion

The current efforts to eliminate the filarial diseases onchocerciasis and lymphatic filariasis and to achieve the United Nations Sustainable Development Goals by 2030 are dependent on the contemporary identification of new treatment options for filariasis. Our study demonstrates that short-course treatment with oxfendazole can achieve sterile cure in the experimental *L*. *sigmodontis* model of filariasis. In this model, oxfendazole is shown to be a selective macrofilaricide with no direct effect against microfilariae, inhibiting filarial embryogenesis and production of microfilariae in female adult filariae. Our *in vivo* and *in vitro* efficacy studies demonstrate that oxfendazole has a time-dependent efficacy. Clinical phase 1 results suggest that reasonable dosing regimens of oxfendazole could be safe at doses we speculate to be efficacious for human filariasis, especially onchocerciasis and potentially lymphatic filariasis and loiasis.

Among the benzimidazole carbamates class, flubendazole has been most widely investigated for activity against filarial nematodes in terms of prophylactic and therapeutic efficacy (summarized in S1 Table). However, flubendazole was originally designed to target gastrointestinal nematodes as a poorly soluble and bioavailable molecule [43]. Consequently, when administered orally, the plasmatic exposure is marginal [44] and therefore efficacy against filarial nematodes is modest [45]. To overcome this limitation, AbbVie and subsequently Janssen Pharmaceuticals, have developed an ASD oral formulation of flubendazole [46] that can reduce the *L*. *sigmodontis* adult worm burden in jirds by 90% after 10 days of treatment [17]. In mice, oxfendazole and flubendazole both have a prolonged but low plasmatic exposure when administered subcutaneously compared to an oral administration. However, oxfendazole has a better efficacy than flubendazole when administered *per os* in a conventional suspension, achieving a complete clearance of the *L*. *sigmodontis* adult worm burden in infected mice. The replacement of the carbonyl group present in flubendazole by a sulfoxide group in oxfendazole probably increases its solubility and therefore oral bioavailability.

The driver for the efficacy of oxfendazole seems to be linked to the treatment duration. *In vitro* efficacy against *O*. *volvulus* pre–adult worms is time dependent, while increasing doses mediate minor improvements in efficacy (Fig 1C). Similarly, *in vivo* efficacy is driven by the treatment duration (Fig 5) and an increase of the area under the curve (AUC) does not improve efficacy of single oral administrations (Fig 5). Thus, efficacy of oxfendazole is consistent with a time dependent effect achieved by maximizing time over a minimum inhibitory concentration.

A comparison of the *in vitro* results indicates that after 120 h exposure to the benzimidazole compounds reduced the motility levels over time for all the *Onchocerca* species tested. At that time point, the compounds are only marginally or moderately active at the concentrations tested and these results are in good agreement with previously documented findings where albendazole, flubendazole and mebendazole showed weak activity against *O*. *gutturosa* or *O*. *volvulus* adult worms *in vitro* [47, 48]. However, the motility of *O*. *volvulus* L5, observed for 28 days, diminished significantly following exposure to oxfendazole for 14 days. A dose response was established and the IC_50_ was reduced by around 2.5 logs from day 14 to 19 (Fig 1C). Immune responses that occur *in vivo* and are missing during *in vitro* experiments may be responsible for the discrepancy of the modest *in vitro* efficacy and good *in vivo* efficacy of the benzimidazoles flubendazole and oxfendazole against filariae.

Interestingly, late embryonic stages are completely absent from recovered *L*. *sigmodontis* adult female worms in jirds treated with a low-dose (2 x 5 mg/kg) of oxfendazole for 10 days. This demonstrates that oxfendazole also suppresses embryogenesis in female worms. This phenomenon was also shown with flubendazole in jirds infected with *L*. *sigmodontis* and histological analyses suggested that flubendazole causes pathological changes in the female worm intestine, body wall, uterus and uterine content, which were arguably defined as permanent [17].

*In vivo* studies of *L*. *sigmodontis* microfilariae-injected mice with oxfendazole showed no relevant impact on microfilariae clearance. Lack of such a biologically relevant microfilaricidal effect at the predicted human efficacious dose (i.e. 2.7 mg/kg) is of great importance for potential treatments of human filarial infections, because some microfilaricidal drugs, such as DEC, are known to cause severe skin inflammation (“Mazzotti reaction”) in endemic areas of onchocerciasis by a rapid killing of skin-dwelling microfilariae [3]. In addition to acute dermatitis, the inflammation caused by rapid killing of microfilariae can cause vision loss in patients with onchocerciasis [1]. Similarly, *Loa loa*-infected individuals with high levels of circulating microfilariae experience severe inflammatory effects, including encephalopathy and death, when treated with microfilaricidal agents [49]. Thus, if oxfendazole lacks microfilaricidal efficacy against human filarial species, safe administration of oxfendazole also in areas co-endemic for onchocerciasis and loiasis could be enabled. A first indication that oxfendazole lacks such a microfilaricidal efficacy against *L*. *loa* was recently provided in the newly established *L*. *loa* mouse model [50].

Our study has several limitations that have to be considered when drawing assumptions for clinical efficacy against human pathogenic filariae. First, inhibition of filarial motility, as shown *in vitro* against *Onchocerca* species in our study, does not subsequently translate to a macrofilaricidal effect, as filarial motility may recover after the effects of the drug fade. However, the motility of *O*. *volvulus* pre-adult L5 worms did not recover after the removal of oxfendazole from the *in vitro* culture, but was further reduced during the following 19 days of washout period, resulting in an IC50 of 1.02·10^−8^ M on day 33 in culture. Next, we used a single *in vivo* filarial rodent model to describe the efficacy of oxfendazole. The *L*. *sigmodontis* rodent model used in this study is a surrogate filarial nematode to model human filarial diseases, as all filarial models currently present have their limitations. New models with immunocompromised mice and the human pathogenic filariae *O*. *volvulus* and *L*. *loa* were recently introduced and infections of jirds with *B*. *malayi* are well established [50–52]. Furthermore, an *Onchocerca ochengi* adult worm jird model was recently developed, confirming the *in vivo* efficacy of oxfendazole against a related *Onchocerca* species [53]. In comparison, the strengths of the *L*. *sigmodontis* model is its development in immunocompetent mice and the development of chronic, patent infections in jirds that allows long-term analysis over several months. Thus, the *L*. *sigmodontis* rodent model was chosen by the Bill & Melinda Gates Foundation for the “Macrofilaricidal Drug Accelerator Program” to identify new compounds with a macrofilaricidal effect [17, 23–25, 54, 55]. Despite this, all filarial rodent models have a shorter filarial life-span in comparison to human pathogenic filariae and differences in the location of the parasites and the associated accessibility of the drug to the filariae (thoracic cavity for *L*. *sigmodontis* versus subcutaneous nodules for *O*. *volvulus*) do exist. However, previous work from our group with doxycycline showed that 4 weeks of doxycycline therapy clear *Wolbachia* endosymbionts and provide a macrofilaricidal efficacy in onchocerciasis and lymphatic filariasis patients [2, 56, 57], demonstrating that the results from the *L*. *sigmodontis* rodent model [27, 58] are transferable to humans in this case. Furthermore, drugs currently used for human filariasis—albendazole, ivermectin and DEC—show a comparable phenotype in the *L*. *sigmodontis* rodent model, clearing microfilariae, but not adult worms. Lack of translation is always a risk when using preclinical screening models. A recent study on the efficacy of oxfendazole highlights this risk of translation, as treatment of *Onchocerca lupi*-infected dogs caused no significant decrease in dermal microfilariae numbers [59]. Lack of efficacy in this study may be explained by an insufficient exposure of the compound in the nodules, by a poor intake by the worms, or by lack of activity against *O*. *lupi*. The latter was not assessed *ex vivo*. Ultimately, the efficacy of oxfendazole for filarial human infections will be determined in upcoming clinical trials.

With the recent progress towards the elimination of lymphatic filariasis, due in part to the introduction of the triple therapy [60], and the partial success of MDA programs for onchocerciasis in highly endemic areas [3], oxfendazole may present a complementary treatment of choice to support elimination in the remaining foci of onchocerciasis and lymphatic filariasis. In particular, when the endemic countries reach lower prevalence of filarial diseases, the cost-effectiveness of community-directed MDA treatments will decrease but short-term treatments with a macrofilaricidal drug could considerably reduce the program timeframes required to reach elimination of both filarial diseases [61, 62]. With the recent proclamation of the United Nations Sustainable Development Goals 2030, which as a consequence calls for the elimination of onchocerciasis and lymphatic filariasis, it is essential to have additional treatment options with macrofilaricidal drugs available until 2025. The macrofilaricidal activity of oxfendazole in the absence of microfilaricidal efficacy observed in the *L*. *sigmodontis* model, renders oxfendazole as a promising lead candidate that should be followed-up as treatment option for filarial diseases.

To support an Investigational New Drug application for oxfendazole as part of the neurocysticercosis trials, a regulatory safety package was conducted [63]. Oxfendazole did not exhibit toxicity *in vitro* in an AMES bacterial assay nor in a mouse lymphoma assay. Subsequent *in vivo* rat micronucleus assays confirmed the *in vitro* results, providing enough assurance for a lack of potential for aneuploidy. These results are in agreement with previously reported data showing that oxfendazole, as known filaria tubulin inhibitor, has a weak affinity to mammalian tubulin as well as poor inhibition of its polymerization [64, 65]. Furthermore, subacute toxicity, behavioral effects in rats and cardiovascular effects in dogs did not raise safety concerns. These results were reviewed favorably by the FDA and led to further evaluation of oxfendazole in a Phase I trial confirming the safety and pharmacokinetics of single oxfendazole administrations in healthy volunteers [20, 42] at doses that are now predicted to mediate macrofilaricidal efficacy. Although results of the multiple ascending dose are not yet published, the design and completion of this study using 3, 7.5 and 15 mg/kg of oxfendazole suggest that dosing for five consecutive days at the predicted human efficacious dose would be possible [https://clinicaltrials.gov/ct2/show/record/NCT03035760?cond=oxfendazole&rank=2].

With the awareness of the risk of translation based on a single preclinical *in vivo* filarial rodent model and limitations of *in vitro* screening assays, our current study supports the consideration of oxfendazole as a macrofilaricidal drug candidate for the treatment of filarial diseases such as onchocerciasis, which could considerably shorten elimination program time frames and help to achieve the Sustainable Development Goals by 2030 with regard to onchocerciasis and if needed also for lymphatic filariasis. We have demonstrated in two independent laboratories that oral and sc treatment regimens of oxfendazole are highly efficacious in clearing *L*. *sigmodontis* adult worms in a dose and treatment duration-dependent fashion. Pharmacokinetic data further highlight that low, but prolonged exposure of oxfendazole mediates this efficacy. Due to its excellent macrofilaricidal efficacy following short-term oral treatment in the *L*. *sigmodontis* rodent model, DND*i* has nominated oxfendazole as a drug candidate for clinical development against human filarial diseases.

## Supporting information

S1 Table Overview of preclinical studies done with benzimidazoles and their efficacy in different animal models of filariasis. (XLSX)

S2 Table Benzimidazoles mediate a moderate inhibition of *O*. *gutturosa* adult worm motility *in vitro*. *Onchocerca gutturosa* parasites were exposed to oxfendazole, albendazole, flubendazole and Immiticide (as a positive control) at the concentrations indicated. *O*. *gutturosa* adult motility values were measured at 24 h intervals up to 120 h. The motility reductions as well as the reduction of the MTT assay are presented as a percentage reduction by comparison to the negative control values.
(DOCX)
