# Supplementary material for: Oxfendazole mediates macrofilaricidal efficacy against the filarial nematode Litomosoides sigmodontis in vivo and inhibits Onchocerca spec. motility in vitro
Source: PLoS Negl Trop Dis. 2020 Jul 6;14(7):e0008427. doi: 10.1371/journal.pntd.0008427 (PMC7365463; doi:10.1371/journal.pntd.0008427)
Supplement: S2 Table — Onchocerca gutturosa parasites were exposed to oxfendazole, albendazole, flubendazole and Immiticide (as a positive control) at the concentrations indicated. O. gutturosa adult motility values were measured at 24 h intervals up to 120 h. The motility reductions as well as the reduction of the MTT assay are presented as a percentage reduction by comparison to the negative control values. (DOCX) [file pntd.0008427.s002.docx]

**Supplementary Table S2:** **Benzimidazoles mediate a moderate inhibition of *O. gutturosa* adult worm motility *in vitro*.** *Onchocerca* *gutturosa* parasites were exposed to oxfendazole, albendazole, flubendazole and Immiticide® (as a positive control) at the concentrations indicated. *O. gutturosa* adult motility values were measured at 24 h intervals up to 120 h. The motility reductions as well as the reduction of the MTT assay are presented as a percentage reduction by comparison to the negative control values.

|  |  | **Motility Reduction (%)** | | | | | **MTT Reduction (%)** |
| --- | --- | --- | --- | --- | --- | --- | --- |
| **Compound** | **Concentration (M)** | **24 h** | **48 h** | **72 h** | **96 h** | **120 h** | **120 h** |
| **Immiticide** | 1.25 x 10^-5^ | 100 | 100 | 100 | 100 | 100 | 91 |
|  | 3.1 x 10^-6^ | 100 | 100 | 100 | 100 | 100 | 94 |
|  | 7.8 x 10^-7^ | 49 | 74 | 93 | 100 | 100 | 79 |
|  | 1.9 x 10^-7^ | 4 | 9 | 33 | 44 | 44 | 51 |
| **Albendazole** | 5.0 x 10^-5^ | 10 | 12 | 29 | 40 | 47 | 47 |
|  | 1.25 x 10^-5^ | 10 | 12 | 27 | 38 | 40 | 28 |
|  | 3.1 x 10^-6^ | 10 | 5 | 16 | 26 | 40 | 19 |
|  | 7.8 x 10^-7^ | 10 | 9 | 13 | 33 | 33 | 18 |
| **Oxfendazole** | 5.0 x 10^-5^ | 16 | 9 | 29 | 33 | 44 | 11 |
|  | 1.25 x 10^-5^ | 10 | 3 | 23 | 31 | 40 | 12 |
|  | 3.1 x 10^-6^ | 10 | 5 | 33 | 31 | 40 | 17 |
|  | 7.8 x 10^-7^ | 4 | 5 | 20 | 31 | 38 | 7 |
| **Flubendazole** | 5.0 x 10^-5^ | 23 | 22 | 36 | 38 | 54 | 20 |
|  | 1.25 x 10^-5^ | 10 | 12 | 33 | 44 | 51 | 2 |
|  | 3.1 x 10^-6^ | 4 | 5 | 16 | 38 | 40 | 3 |
|  | 7.8 x 10^-7^ | 6 | 9 | 13 | 33 | 26 | 3 |
